# Adult traumatic Bochdalek hernia discovered a decade after injury

**DOI:** 10.11604/pamj.2026.53.67.50310

**Published:** 2026-02-09

**Authors:** Shubhada Sangameshwar Kadadi, Mohammad Arshad Ansari

**Affiliations:** 1 Department of Emergency Medicine, Jawaharlal Nehru Medical College, Datta Meghe Institute of Higher Education and Research, Wardha, India

**Keywords:** Bochdalek´s hernia, surgical emergency, traumatic

## Image in medicine

A 60-year-old man presented to the emergency department with an 8-day history of progressive breathlessness. He reported a head-on motor vehicle collision 10 years earlier but remained asymptomatic and was never evaluated at that time. Clinical examination revealed markedly reduced air entry over the left hemithorax, with audible bowel sounds in the same region. Chest radiograph (A) and high-resolution computed tomography (HRCT) thorax (B) demonstrated a large ~5.8 cm posterolateral defect in the left hemidiaphragm, consistent with a Bochdalek hernia, with herniation of the stomach and small bowel loops into the left thoracic cavity. Associated rightward mediastinal shift and mild compressive atelectasis of the left lung were noted. The patient underwent prompt surgical repair with complete relief of symptoms. This case highlights the need for thorough evaluation following thoracoabdominal trauma-even in asymptomatic individuals-and reinforces that delayed presentation of traumatic Bochdalek hernia, though rare in adults, is a surgical emergency due to the risk of incarceration and strangulation.

**Figure 1:**
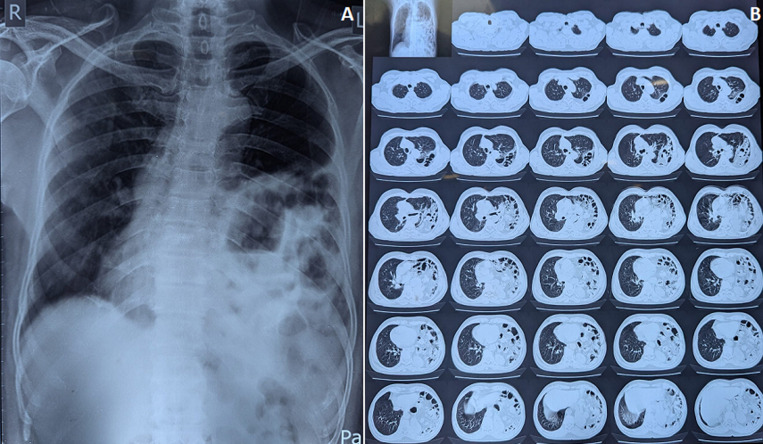
A) chest X-ray showing herniation of the stomach and small bowel loops into the left thoracic cavity with rightward mediastinal shift; B) high-resolution computed tomography of the thorax showing ~5.8 cm posterolateral defect in the left hemidiaphragm

